# Administration of Ethanolic Extract of *Spinacia oleracea* Rich in Omega-3 Improves Oxidative Stress and Goblet Cells in Broiler Chickens Infected with *Eimeria tenella*

**DOI:** 10.3390/molecules28186621

**Published:** 2023-09-14

**Authors:** Osama Ewais, Heba Abdel-Tawab, Huda El-Fayoumi, Shawky M Aboelhadid, Saleh Al-Quraishy, Piotr Falkowski, Abdel-Azeem S. Abdel-Baki

**Affiliations:** 1 Department of Parasitology, Zoology Department, Faculty of Science, Beni-Suef University, Beni-Suef 62511, Egypthoba_abdo_2010@yahoo.com (H.A.-T.); aabdelbaki@science.bsu.edu.eg (A.-A.S.A.-B.); 2 Parasitology Department, Faculty of Veterinary Medicine, Beni-Suef University, Beni-Suef 62511, Egypt; 3 Zoology Department, College of Science, King Saud University, Riyadh12211, Saudi Arabia; squraishy@ksu.edu.sa; 4 Department of Epizootiology and Clinic for Birds and Exotic Animals, Faculty of Veterinary Medicine, Wroclaw University of Environmental and Life Sciences, pl. Grunwaldzki 45, 50-366 Wrocław, Poland; fal.wet@outlook.com

**Keywords:** spinach, emeriosis, omega-3, histological, oxidative stress

## Abstract

This study investigated the anticoccidial activity of spinach (*Spinacia oleracea*) whole-plant extract against *Eimeria tenella*, both in vitro and in vivo. For this purpose, one hundred 8-day-old broiler chicks of both sexes were divided into four groups (*n* = 25 in each group). Chicks in the first group served as the negative control (non-treated–non-infected). Chicks in the second group were challenged at 18 days old with 5 × 10^4^ *E. tenella* sporulated oocysts. The third group was challenged with 5 × 10^4^ sporulated *E. tenella* oocysts at 18 days old after receiving spinach extract at a dose of 50 mg/kg at 8 days old. The fourth group received 0.2 mg/kg diclazuril (Coxiril^®^ 0.2%) in their diet two days before being orally infected with 5 × 10^4^ sporulated *E. tenella* oocysts and this continued till day 10 post-infection (PI). The growth performance, clinical symptoms, oocyst shedding, histological findings, and biochemical parameters were used to evaluate the efficacy on day 8 PI when the infection was at its peak. A gas chromatography examination revealed that omega-3 fatty acids were the main constituents of the spinach extract, followed by oleic acid, palmitic acid, and phytol, with amounts of 23.37%, 17.53%, 11.26%, and 7.97%, respectively. The in vitro investigation revealed that the spinach extract at concentrations of 10% and 5% inhibited the oocyst sporulation by 52.1% and 45.1%, respectively. The 5% concentration was selected for the in vivo trial based on the results of the in vitro study. The infected–untreated group showed high levels of OPG; lower body weight; a greater number of parasite stages; few goblet cells; decreased SOD, CAT, and GPX levels; and increased MDA and NO levels. The spinach-treated group, on the other hand, showed a significant decrease in oocyst output per gram of feces (OPG), increased body weight, decreased parasitic stages, and a nearly normal number of goblet cells. Additionally, it reduced malondialdehyde (MDA) and nitric oxide (NO), while increasing superoxide dismutase (SOD), catalase (CAT), and glutathione peroxidase (GPX). In conclusion, spinach produced significant antioxidant effects, increased body weight, reduced the number of oocysts and parasite stages in the caecum, and restored the number of goblet cells relative to those of an uninfected control. Furthermore, spinach extract inhibits the sporulation *percentage* of *E. tenella* oocysts. The ethanolic extract of *S. oleracea* (whole plant) contained high concentrations of fatty acids, palmitic acid, Phytol, betulin, and ursolic aldehyde, all of which are known to regulate the antioxidant pathway and modulate inflammatory processes and may be the main reason for its anticoccidial activity.

## 1. Introduction

Members of the genus *Eimeria* are the most frequent parasites that infect chickens [1,2,3]. *Eimeria* is an apicomplexan parasite that comprises several species and is the causative agent of coccidiosis. Coccidiosis is common wherever chickens are raised (industrial, traditional, or organic/bio farms), and it remains one of the main illnesses that have a negative impact on bird performance in intensive production systems because it causes intestinal lesions (such as inflammation, diarrhea, and hemorrhage), growth impairment, poor feed utilization, non-homogenized flock weights, and increased mortality [4]. Avian coccidiosis causes enormous economic losses in poultry production worldwide [1]. According to estimates made by Rashid et al. [2], there are losses in a variety of poultry categories that vary from USD 104.74 to USD 2,750,779.00 due to the expense of the vaccine, treatment, and other preventative measures.

There are several drugs available to treat coccidiosis; however, their overuse has hastened the development of multidrug resistance and increased the residue in tissues. As a result, a global strategy is being developed to explore the antiparasitic capabilities of various herbal plants in order to alleviate the problems caused by coccidial illness in the chicken industry. The main advantages of using herbal-based therapies to treat coccidiosis are their low toxicity and inexpensive production costs [3]. Spinach (*Spinacia oleracea*) belongs to the family Amaranthaceae and is a highly significant and nutrient-dense plant. It contains numerous amounts of B vitamins, riboflavin, foliate, niacin, soluble dietary fiber, omega-3 fatty acids, and minerals. Spinach is also abundant in iron, which protects against iron-deficiency-related illnesses, such as osteoporosis and anemia [4]. Spinach contains a variety of active compounds, including flavonoids and other polyphenolic active ingredient molecules that act as antioxidants and anti-inflammatory agents. When ingested together, the flavonoids, carotenoids, vitamins (C, E), and phenolic components in spinach have an antioxidative effect that reduces the harmful effects of free radicals [5]. In addition, spinach leaf extract and its phytoconstituents were shown to have anti-inflammatory, antidiarrheal, antibacterial, antioxidant, and insecticidal properties [6]. This study therefore aimed to investigate the anticoccidial effect of spinach extract on *Eimeria tenella* in experimentally infected broiler chicks.

## 2. Results

### 2.1. GC-MS of Spinach Extract

**Figure 1:**
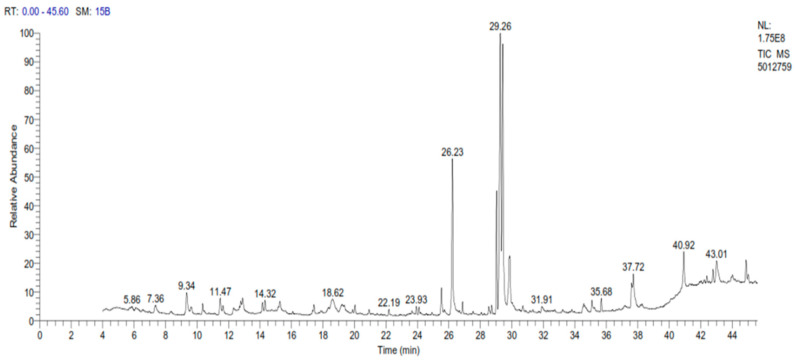
*GC-MS* chromatogram of spinach extract.

A sample of spinach extract was subjected to gas chromatography examination, which revealed a high number of elements, with omega-3 accounting for the greatest amount, followed by oleic acid, palmitic acid, and phytol with concentrations of 23.37%, 17.53%, 11.26%, and 7.97%, respectively, as shown in Table 1 and Figure 1.

### 2.2. In Vitro Studies of Spinach Extract on Eimeria tenella Oocysts

**Figure 2:**
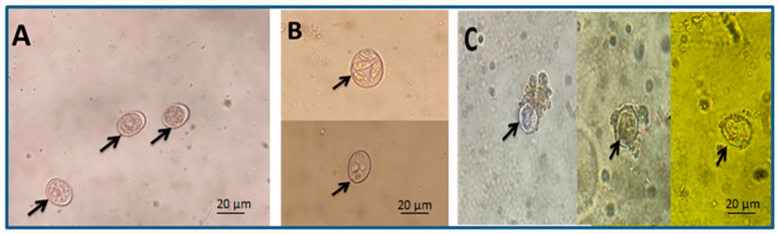
Arrows refer to different oocyst shapes: (**A**) unsporulated oocysts, (**B**) sporulated oocysts, and (**C**) damaged oocysts (scale bar—20 μm, seen with a 40× lens).

Using 10% and 5% spinach extracts achieved an inhibition of oocysts sporulation at rates of 52.1% and 45.1%, respectively, and the lowest concentration (0.625%) caused a sporulation inhibition percentage of 10.27% after 72 h (Figure 2). Meanwhile, 10% formalin (positive control) caused an estimated inhibition of 67.27% (Table 2).

### 2.3. Bloody Diarrhea

All infected groups were observed to have bloody diarrhea, but its severity varied from the highest in the positive control group, then the diclazuril group, to the lowest in the spinach group. The highest degree of bloody diarrhea in all infected groups was noticed on the fifth DPI and the spinach extract achieved a lower degree of bloody diarrhea than diclazuril (Table 3).

### 2.4. Survival Percentage and Lesion Scoring

It was noticed that the treated groups had a higher survival percentage than the infected–untreated group and the lesions had a low severity in the treated groups compared with the untreated group. In the diclazuril group, the number of deaths was lower than in the spinach group. Mild lesions were recorded in the diclazuril and spinach groups, but there were severe lesions in the infected–untreated group (Table 4).

### 2.5. Oocysts per Gram of Feces

Fecal samples were collected from all groups for analysis from the 4th to the 10th DPI. The number of oocysts increased until they reached peaks in the diclazuril and spinach groups on the 8th DPI, achieving 3819 and 7906 oocysts/g, respectively, and then dramatically decreasing, achieving totals of 7504 and 18,894, respectively, after 10 days post-infection with *Eimeria tenella* oocysts; the values in the positive control group continued to increase until they reached the peak on the 9th DPI with 58,696 oocysts/g and achieved a total of 92,263 oocysts/g after 10 days post-infection (Table 5).

### 2.6. Oocyst Index

**Figure 3:**
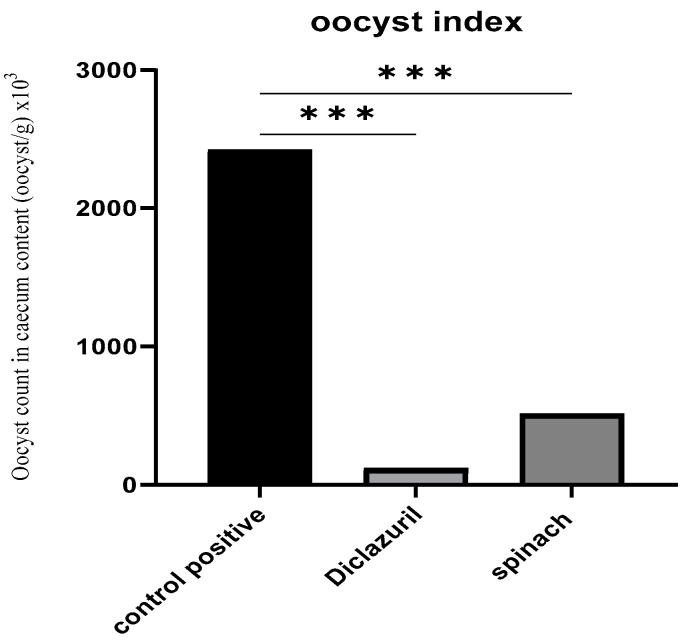
Oocyst index of caecal content (oocyst/g). (***) The mean difference was significant at *p* < 0.001.

Following the examination of the cecum’s contents and the counting of the oocysts, it was found that the treated groups had a decreased number of oocysts by about 95.1% and 78.8% for the diclazuril group and spinach group, respectively, compared with the positive control (Figure 3).

### 2.7. Feed Conversion Ratio

During the first 2 weeks of age, all groups had a close FCR because the infection had not occurred yet, but at the end of the third week, differences began to appear after infection on the 18th day of age: a significant difference was noticed between the untreated group and treated groups and the conversion rate was greater than the untreated groups. The results for all groups with *p*-values compared with the positive control are shown in Table 6.

### 2.8. Histopathological Studies

#### 2.8.1. Number of Developmental Parasitic Stages and Goblet Cells in Ceca of Chickens

There was a significant difference in the number of parasitic stages and the number of goblet cells in the treated groups relative to the infected–untreated groups. The diclazuril group had a lower number of parasitic stages than the spinach group but the spinach group contained a higher number of goblet cells than the diclazuril group (Table 7).

**Figure 4:**
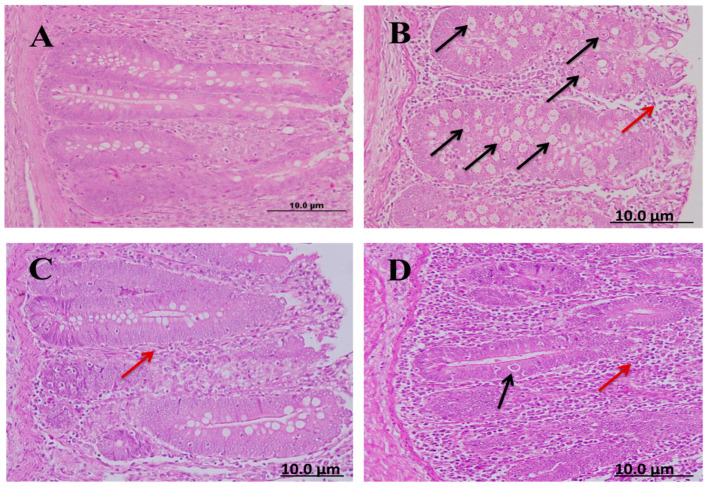
Cecal sections of chickens in different groups showing the number of parasitic (H&E); (**A**) negative control group (noninfected–untreated), (**B**) positive control group (infected–untreated), (**C**) Diclazuril group (infected–treated with diclazuril 0.2 g/kg of food), and (**D**) spinach group (infected–treated with spinach extract 50 mg/kg of body). Black arrows refer to parasitic stage while red arrows refer to leukocytic infiltration.

Cecal sections of different groups were collected on the 8th day after infecting chickens with 50,000 sporulated *Eimeria tenella* oocysts; the sections were stained with hematoxylin and eosin stain to determine the number of parasitic stages. It was found that the diclazuril group had a lower number of parasitic stages than the spinach group (Figure 4).

#### 2.8.2. Number of Goblet Cells in Ceca of Chickens

**Figure 5:**
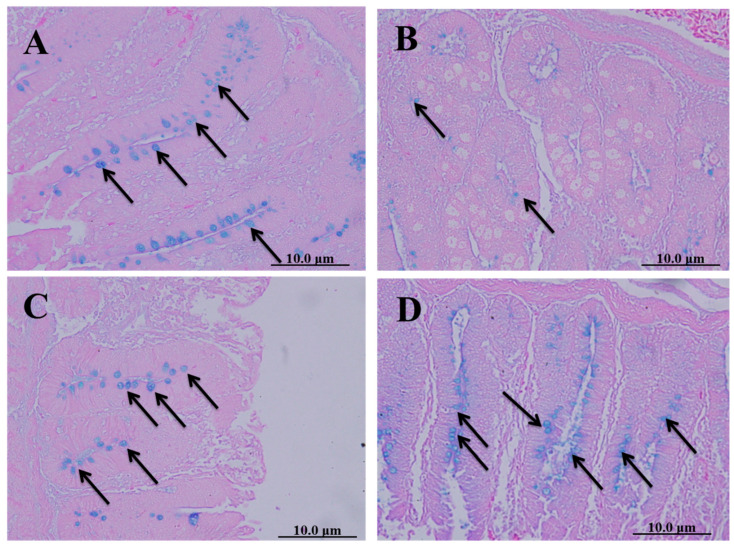
Cecal sections of chickens in different groups stained with alcian blue stain to determine the number of goblet cells. (**A**) Negative control group (noninfected–untreated), (**B**) positive control group (infected–untreated), (**C**) Diclazuril group (infected–treated with diclazuril 0.2 g/kg of food), and (**D**) spinach group (infected–treated with spinach extract 50 mg/kg of body weight). Black arrows refer to goblet cells.

Cecal sections of different groups were collected on the 8th day after infecting chickens with 50,000 sporulated *Eimeria tenella* oocysts; they were stained with alcian blue stain to determine the number of goblet cells. The spinach extract achieved a better effect than diclazuril in returning the number of goblet cells close to normal (Figure 5).

### 2.9. Biochemical Studies on the Ceca of Chickens Infected with Eimeria tenella

**Figure 6:**
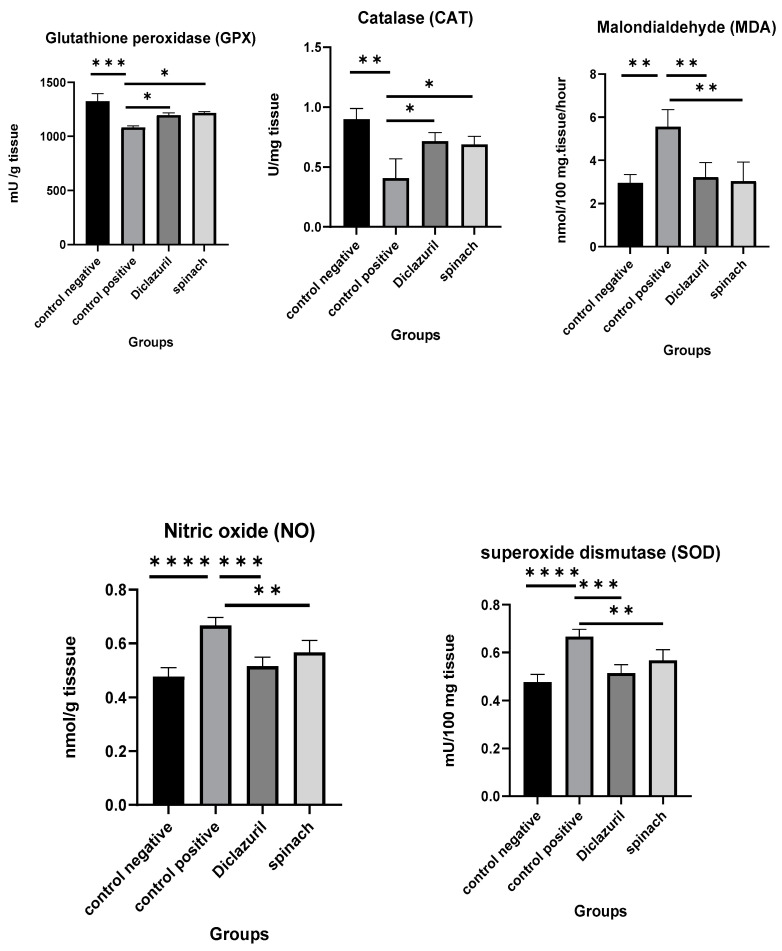
Effect of diclazuril and spinach extract on levels of some oxidative stress markers in ceca of chickens in different groups. (*) the mean difference is significant, (**) Low significance, (***) moderate significance, and (****) very high significance at *p*-value < 0.05.

The effects of diclazuril and spinach extract on the levels of some oxidative stress markers in the ceca of chickens that were infected with 50,000 sporulated *Eimeria tenella* oocysts on the 18th day of age and then humanly killed on the 8th DPI was measured and compared with the positive control (infected–untreated). It was obvious that the diclazuril and spinach groups contained higher levels of GPX, CAT, and SOD than the positive control and lower MDA and NO than the positive control. This indicates that the spinach extract had a good effect on preserving cells from oxidative stress. There was no significant difference between the diclazuril and spinach groups (Figure 6).

## 3. Discussion

Polyether ionophore antibiotics, chemically synthesized anticoccidial drugs, and vaccines are the main methods of preventing and controlling coccidiosis in chickens. However, resistance has developed for all of the drugs currently in use, which remains a significant limitation for their use [7]. Although vaccination is a viable alternative to the synthetic chemical anticoccidial drugs, its use is constrained by a laborious manufacturing process, the high expense of production and licensing of new vaccines, and the danger of pathogen spread (with the injection of attenuated coccidiosis vaccines) [8,9]. Therefore, interest in research on alternate anticoccidial treatments, such as plant extracts, has been substantially developed [10,11]. Following this approach, the current study compared the in vitro and in vivo efficacies of spinach extract and diclazuril against *Eimeria tenella* in experimentally infected chickens.

The *GC-MS* technique was employed in this investigation to determine the probable chemical compounds in an extract of *Spinacia oleracea*. According to the findings of this study, the ethanol extract of *S. oleracea* (whole plant) contained a high concentration of fatty acids, sterol, diterpenoids, and triterpenes, all of which are known to regulate the antioxidant pathway and modulate inflammatory processes with anti-cancer properties [12,13,14]. Similar components were reported by Abdelgawad et al. [15] in their phytochemical analysis of *S. oleracea* leaves from Egypt. According to the gas chromatography analysis, omega-3 made up the largest proportion of the spinach extract, which is consistent with the findings of Hetta et al. [16], who found that omega-3 is a major component of *S. oleracea* flowers (30.53%). Similar to our findings, it was found that the main fatty acids in *Polyalthia longifolia* seed oil were oleic, linoleic, and palmitic acids. It was also found that these acids had several biological properties, including anti-lipooxygenase, antioxidant, anti-inflammatory, anti-parasite, anti-microbial, and cytotoxicity properties. α-linolenic acid (ALA), which is an omega-3 fatty acid, and Linoleic acid (LA), which is an omega-6 fatty acid, are regarded as essential fatty acids that are known to have antibacterial properties [17]. Therefore, the South African plants *Helichrysum pedunculatum* (family: Asteraceae) and *Schotia brachypetala* (family: Fabaceae), which mostly consist of linoleic and oleic acids as main components, are used for wound healing [18,19]. C18 fatty acids, including oleic, linolenic, and linoleic acids, were demonstrated to have antimalarial effects by preventing the proliferation of malarial parasites in mice infected with *Plasmodium vinckei petteri* and *Plasmodium yoelii nigeriensis* [20]. Also, omega-3 fatty acids were found to provide health benefits in the treatment of depression and schizophrenia [21,22], as well as benefits for cancer, inflammatory bowel disease, rheumatoid arthritis, psoriasis, and mental health [23]. In addition, omega-3 polyunsaturated fatty acids (ω-3PUFAs) are hypothesized to have beneficial effects on reducing cytokines and inflammation-associated proteins by altering the signaling pathways that regulate gene expression in inflammatory cells [24]. One example of these pathways is nuclear factor kappa B (NF-B), which is linked to the activation of the genes encoding several cytokines, adhesion molecules, and COX-2 [25]. The proliferator-activated receptor (PPAR), which is activated by ω-3PUFAs, is an anti-inflammatory transcription factor that prevents NF-B from entering the nucleus. Allen et al. [26] evaluated the effects of ω-3PUFAs from animal or plant sources on cecal lesions caused by *Eimeria tenella* (80 strain) in chicken vs. medium chain triglycerides (MCTs) or a simple starter diet (SS). They found significant decreases in the mean cecal lesion scores, as well as decreases in bird mortality and significant increases in IL-6 blood levels. Choi et al. [27] discovered that ω-3PUFAs increased autophagy activation, resulting in decreased intracellular survival of *T. gondii*, suggesting that ω-3PUFAs could be used as a therapeutic candidate to prevent toxoplasmosis and infection with other intracellular protozoan parasites. According to Stok and Francis [28], the unsaturated fatty acid oleic acid was shown to be able to inactivate influenza type A virions. It was found that supplementation with omega-9, or oleic acid, is crucial in the metabolic dysfunction that occurs during sepsis because it increases the levels of the anti-inflammatory cytokine IL-10, lowers levels of the pro-inflammatory cytokines TNF- and IL-1, and inhibits neutrophil migration [29].

Through in vitro testing, it was discovered that the highest concentration of spinach extract produced the highest percentage of oocyst sporulation inhibition. This may have been due to the ability of the spinach extract to modify the permeability of the oocyst wall, penetrate the oocyst wall, and damage the oocyst’s contents, as indicated by the cracked oocysts subjected to 10% spinach extract. Molan et al. [30] found similar effects when they tested pine bark (*Pious radiata*, family: Pinaceae) extract on the sporulation of three species of avian coccidian oocysts. Fatemi et al. [31] also found that *Artemisia annua* (family: Asteraceae) extract has a significant effect on the sporulation rate of mixed oocysts of *Eimeria acervulina*, *Eimeria necatrix*, and *Eimeria tenella*. The number of oocysts per gram of feces and cecal content of oocysts was reduced in the spinach-treated chicks. It was discovered that spinach extract has a high concentration of phytonutrients, such as fatty acids, palmitic acid, Phytol, betulin, and ursolic aldehyde [32]. It was proposed that these compounds may change the permeability of the cytoplasmic membrane, which inhibits many physiological functions and results in a loss of membrane potential, allowing vital cellular components to leak out, inhibiting protein and ATP production, and causing cellular death [33,34,35]. The present study found that following infection with *E. tenella*, the infected chicks had a higher cecal lesion score and less weight gain than the chicks in the negative control group similar to those reported by Conway et al. [36]. According to Christaki et al. [33], the decrease in body weight in *E.*-*tenella*-infected chicks was attributed to the infection’s negative effects on food digestion, absorption, and metabolism. The spinach-treated chicks recovered rapidly from infection and displayed a low degree of lesions because spinach contains numerous potent chemicals, including phytol and palmitic acid, which can accelerate and enhance wound healing [37]. Additionally, phytol has been found to possess potential anti-schistosome, antinociceptive, antioxidant, anti-inflammatory, and antiallergenic properties. Phytol is a frequently used food additive and serves as a precursor in the synthesis of vitamins E and K. Also, phytol possesses antibacterial properties that are effective against *Staphylococcus aureus* and *Mycobacterium tuberculosis* [38]. Vitamin K has been used to treat hemorrhages associated with coccidiosis and other diseases due to its coagulation-promoting properties [39,40].

The body weight of the chicks in the spinach group significantly increased. Similarly, Wagde et al. [41] found a significant increase in the body weight of ornamental fish (*Xiphophorus hellerii*) fed a spinach-extract-supplemented diet. These findings are in agreement with those of Abbas et al. [42], who noted a marked increase in body weight in the chicks treated with turmeric compared with the infected-untreated group. The diclazuril-treated group exhibited a significant increase in body weight and a decrease in oocyst count; this finding was also consistent with that of Abbas et al. [43].

Because spinach extract is high in fatty acids, it may have an inhibitory effect on different intracellular developmental stages of *Eimeria*, similar to their effect on *Giardia duodenalis* in vitro, which is severely impacted by media supplemented with fatty acids [44]. The two primary immune-competent cells in the intestine are known to be mast cells and goblet cells. According to some studies, the mucus that goblet cells secrete can serve as a protective barrier [45]. Mucus serves as the first line of defense against infections by protecting the gut epithelial layer from pathogens [46]. The decrease in goblet cells may indicate damage to the stem cell population. Goblet cells are produced through mitosis by multipotent stem cells located close to the base of the crypt [47]. Changes in the number of goblet cells can hinder the parasite-infected host’s ability to prevent opportunistic infections from spreading or penetrating the local epithelium [48]. The increase in goblet cells after infection may be due to the active compounds in spinach due to its strong anticoccidial properties and capacity to modify the goblet cell response after infection. This result is similar to that reported by Ramirez et al. [49], who found that neem increased the number of goblet cells in the mouse jejunum after infection with *E. papillata*. Our results suggest that spinach may be useful as a food supplement for animals with an Eimeria infection.

Spinach extract also contains ursolic aldehyde and betulin which have antiparasitic properties against *Trypanosoma cruzi*, as indicated by [50,51].

Spinach extract was discovered to have a powerful effect on free radical reduction because it includes palmitic acid and phytol, both of which have a substantial antioxidant effect in trapping free radicals [25,52,53]. Their antioxidative properties are enhanced by suppressing reactive oxidant species (ROS) accumulation while recovering antioxidant enzymes, including superoxide dismutase and catalase [53]. Several studies indicated that omega-3 PUFAs are essential for improving broiler immunity [54,55]. Highly unsaturated omega-3 fatty acids can permeate the parasite’s tissues. After invasion, they are more vulnerable to oxidative assault by phagocytic cells. This oxidative stress has a deleterious impact on coccidian development [56,57]. Oxidative stress, which is hypothesized to be caused by highly unsaturated omega-3 fatty acids, is likely to be deleterious to coccidia growth. According to certain theories, the membranes of coccidia contain unsaturated fatty acids. During development, their membranes undergo continual turnover, and dietary fatty acids influence the composition of the membranes. There, free radical-producing leucocytes will attack the coccidia, making them more vulnerable to oxidation [56,57].

## 4. Materials and Methods

This experiment was approved by the Beni-suef University Institutional Animal Care and Use Committee (BSU-IACUC), and the approval number is (022-237). Ethanol 70%, formalin, potassium dichromate, and phosphate buffer saline were among the chemicals used in this experiment; they were purchased from PIOCHEM chemical company in 6 October City, Egypt, while Diclazuril (Coxiril ^®^ 0.2%) was obtained from Huvepharma N.V. Uitbreidingstraat 80, 2600 Antwerp, Belgium.

### 4.1. Botanical Extract Preparation

Spinach (whole plant) was purchased from a local market and identified by specialists in the Botany Department, Faculty of Science, Beni-suef University, Egypt. The ethanol leaf extract of spinach was prepared according to Okechukwui et al. [58] with some modifications. Briefly, the plant was cleansed with distilled water and air dried for one week before being ground into powder in an electrical blender. This powder was soaked in 70% alcohol for three days at room temperature in a ratio of 1:2 *w*/*v* (plant powder to 70% alcohol). During this period, the mixture was agitated three times per day. Finally, it was filtered through filter paper, and the filtrate was concentrated and dried in a rotatory evaporator at 40–50 °C. A total of 15 g of greenish viscous extract was produced for every 100 g of spinach powder. The extracted material was kept in the refrigerator at 4 °C until usage, as phytochemicals’ bioavailability is impacted by the matrix and microstructure of the food they are present in, as well as the storage circumstances and temperature range [59]. Cold storage at temperatures below 5 °C is recommended for spinach to maintain its quality and improve its shelf life [60].

#### Gas Chromatography–Mass Spectrometry (*GC-MS*) of Spinach Extract

The spinach was analyzed at the Nawah Scientific Educational Research Centre in Egypt “(https://nawah-scientific.com/) accessed on 26 September 2023” using a Trace Ultra gas chromatograph (GC) combined with a Thermo Scientific DSQ II mass spectrometer (MS). The chemicals were separated on a TR-5MS (30 m × 0.25 mm × 0.25 m) capillary column (Thermo Scientific) with helium flowing at a rate of 1 mL/min and a temperature controlled from 60 to 250 °C at 3 °C/min. Temperatures of 220 and 250 °C were chosen for the MS transfer line and injector, respectively. One milligram of spinach extract was diluted in one milliliter of acetone to make the sample. Manual splitless injection of the diluted material was performed in a volume of one liter. Mass spectra were acquired with the ion source temperature set at 240 °C and the MS set to EI mode at 70 eV. The compounds’ relative retention indices and mass spectra were compared with relevant information in the literature and databases [61]. The relative retention index (RRI) was constructed using a series of n-alkanes (C8–C24). The relative percentages of the chemicals were determined through the use of area percentage data.

### 4.2. Eimeria tenella Oocysts Collection

The coccidian oocysts of *Eimeria tenella* were collected from naturally infected chicks. The oocysts were concentrated, sporulated in 2.5% potassium dichromate, and then orally inoculated in five healthy chicks to propagate the oocysts [62]. Eight days post-inoculation, the birds were euthanized and the cecal contents were obtained. The oocysts were concentrated, then sporulated as previously mentioned, and stored in a refrigerator (2–5 °C) until use [63].

### 4.3. In Vitro Evaluation of Spinach Extract against E. tenella Oocysts

In a 96-well ELISA plate, the anticoccidial activity of spinach extract against *E. tenella* oocysts in vitro was evaluated. The extract was dissolved in potassium dichromate 2.5% (*w*/*v*) to prepare five concentrations of the extract: 10%, 5%, 2.5%, 1.25%, and 0.625. Each concentration was then placed in a well and 1200 oocysts were added. Then, 2.5% potassium dichromate was used as a negative control and 10% formalin served as the positive control. Each concentration was used in three replicates. The plate was incubated at 30 °C and after 24, 48, and 72 h, 25 µL from each well was placed on a glass slide and inspected under a microscope to determine the percentages of sporulated oocysts [64]. The sporulation inhibition effect of the extract was then estimated using the formula: (% of inhibition = sporulation% of negative control − sporulation% of spinach extract). The concentration of spinach extract that had the greatest effect was utilized again to examine its effect in vivo.

### 4.4. Experimental Design of In Vivo Evaluation of Spinach Extract against Eimeria tenella Oocysts on Broiler Chickens

**Figure 7:**
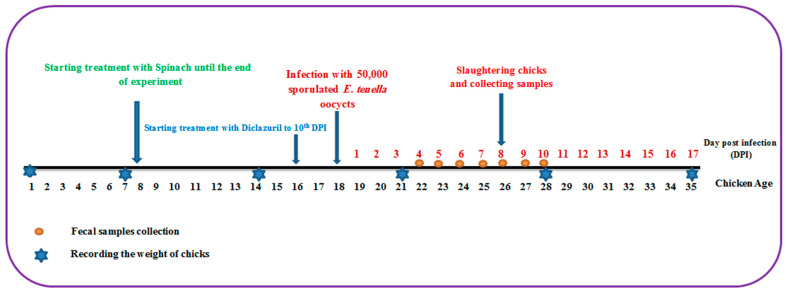
The experimental design depicts the entire course of the experiment, from the first day to the end.

One hundred one-day-old broiler chicks of both sexes were purchased from El-Abd local company. These chicks were reared in wire-mesh batteries with free access to water and food, and the temperature was set at 34 °C, then decreased by 2 °C per week. At the age of 8 days, these chicks were divided into four groups of 25 chickens each as follows: The first group comprised uninfected chicks and served as the negative control group. The second group was infected orally with 5 × 10^4^ *E. tenella* sporulated oocysts at the age of 18 days and acted as the infected control group. The third group was the spinach-treated group, which began receiving spinach extract at a dose of 50 mg/kg supplemented with water at the age of 8 days as a prophylactic treatment and continued until the end of the experiment while being orally infected with 5 × 10^4^ sporulated oocysts of *E. tenella* at the age of 18 days and continuing the spinach treatment. The fourth group was the drug-treated group, which received 0.2 mg/kg diclazuril (Coxiril ^®^ 0.2%) in food two days before being orally infected with 5 × 10^4^ *E. tenella* sporulated oocysts till day 10 post-infection (PI). At day 8 PI, five chickens from each group were sacrificed and ceca were retrieved for further studies (Figure 7).

### 4.5. Evaluation of Spinach Extract Anticoccidial Activity

#### 4.5.1. Clinical Examination, Clinical Symptoms, Mortality, and Bloody Diarrhea

The chicks were examined daily for clinical signs, such as anorexia, ruffled feathers, diarrhea, crowding, loose droppings, difficulty breathing, and bloody droppings. The number of deaths was also recorded on a daily basis. From day 4 to day 10 PI, fecal samples were collected from each group and grossly inspected for blood.

#### 4.5.2. Lesion Scoring and Cecal Core

On day 8 PI, five birds were randomly selected from each group and inspected for lesion scoring. The 0–4 lesion-scoring categorization was used to classify the lesions, which included petechial hemorrhages, bloody fecal contents, cecal wall thickening, and mucoid discharge. Based on the severity of the lesions, no lesions (0), mild lesions (1), moderate lesions (2), severe lesions (3), or very severe lesions (4) were determined for each bird [65].

#### 4.5.3. Parasitological Examination (Oocyst Count per Gram of Feces)

From day 4 PI to day 10 PI, randomly selected fecal samples from each group were collected and prepared for examination according to the methods indicated by Hodgson [66] and Long and Rowell [67]. In brief, 10 g of feces were dissolved in 100 mL of water and left at room temperature in well-covered cups for 24 h. After carefully mixing and sieving the solution, 15 mL of it was centrifuged for 5 min. After removing the supernatant, the pellet was resuspended in a few milliliters of saturated salt solution (NaCl) using a vortex or by tapping the tube. More salt solution was added to the initial 15 mL volume, and the tube was repeatedly turned upside down. Once a sample was added to the McMaster counting chamber by a Pasteur pipette, oocysts floated to the top of the solution, the number of oocysts was counted, and the total number of oocysts per gram was estimated using the equation below:

$$\text{Number of oocysts per gram of litter}=\frac{n}{0.15}\times volume\times 0.1$$

where “*n*”—number of counted oocysts, “0.1”—correction for 10 g originally taken from the litter, “0.15”—volume of the McMaster counting chamber, and “*Volume*”—100 mL of water in which the litter was soaked.

#### 4.5.4. Oocyst Index

The ceca of the five chicks previously selected on the day 8 PI were dissected and their contents were collected and prepared for counting their oocyst content using the previously mentioned McMaster chamber method [66,67,68].

### 4.6. Growth Performance

The chicks were weighed once a week from the start of the experiment to the end. The weekly increase in body weight was calculated by subtracting the starting weight from the total weight. From day 1 to day 20, the birds were fed a coccidiostat-free commercial starter food. When the birds were 21 days old, the starting diet was replaced with a finisher diet, which was fed till the end of the experiment. During the pre-infection and post-infection periods, daily feed and water intake were recorded. The total feed intake for each week was calculated by deducting the amount of feed rejected from the feed supplied. Finally, the feed conversion ratio (FCR) was determined for each week using this formula: $FCR=\frac{\text{feed intake}}{\text{weight increase}}$.

### 4.7. Histological Studies

Parts of the ceca from the previously selected five chicks on day 8 PI were separated and fixed in 10% phosphate-buffered formalin. Then, these samples were processed for normal histology, as described by Tanweer et al. [65]. Sections were stained with hematoxylin and eosin (H&E) to estimate the number of parasitic stages while other sections were stained with Alcian blue to assess the number of goblet cells. The number of goblet cells in each animal’s cecum was counted in well-oriented villous crypt units (VCUs). The results were expressed as the average number of goblet cells per 10 VCUs [69]. In addition, H&E sections were examined for inflammation, necrosis, degeneration, and other pathological alterations.

### 4.8. Biochemical Studies (Oxidative Markers) on the Ceca of Infected Birds

Parts of the selected ceca were washed in 10% phosphate-buffered solution before being homogenized with 0.9% saline at a 1:10 g/mL ratio and centrifuged at 2000 rpm for 10 min. The resultant supernatant was separated, divided into aliquots, and stored at −80 °C in eppendorf tubes to determine some oxidative stress markers. The level of lipid peroxidation was determined by measuring the amount of malondialdehyde (MDA) produced by the breakdown of polysaturated fatty acids. The interaction of lipid peroxidation products with thiobarbituric acid quantifies the products of lipid peroxidation [70]. Superoxide-Dismutase (SOD) activity was determined using pyrogallol autoxidation inhibition as described by Marklund and Marklund [71]. Peroxidase activity (GPX) was measured spectrophotometrically according to the procedure suggested by Manoranjan and Mishra [72], catalase (CAT) activity was estimated in the tissue extract according to the method described by Cohen et al. [73], and the nitric oxide (NO) concentration was determined using the method outlined by James and Glaven [74].

### 4.9. Statistical Analysis

Data was statistically analyzed using SPSS version 17.0 and graph pad prism 8 software. One-way ANOVA, followed by least significant difference (LSD) was used for the comparison between the test and control group and data are expressed as the mean ± standard deviation (SD); *p*-values <0.05 (*p* < 0.05) were considered statistically significant.

## 5. Conclusions

Spinach has an anticoccidial impact because it inhibits the sporulation percentage of *Eimeria tenella* oocysts, increases body weight, decreases the count of oocysts and parasitic stages in the cecum, restores the number of goblet cells to that of an uninfected control, and has a substantial antioxidant effect. In the present work, we used 5% spinach extract in vivo because it had a high sporulation inhibition effect relative to 10% spinach extract in vitro, but we recommend using higher concentrations to study its effect on coccidiosis models and other species that infect chickens and other animals.

## Figures and Tables

**Table 1:** Chemical composition of the spinach extract.

	Compound Name	RT (Min)	Area (%)	MF	Molecular Formula	Molecular Weight (g)
1	Alpha-Linoleic acid (omega-3)	29.26	23.37	909	C_15_H_32_O_2_	280
2	Oleic Acid (omega-9 fatty acid)	29.42	17.53	931	C_18_H_34_O_2_	282
3	Palmitic acid	26.23	11.26	931	C_16_H_32_O_2_	256
4	Phytol (present in vitamin K, vitamin E, and other tocopherols)	29.03	7.97	930	C_20_H_40_O	296
5	Ethyl linolenate	29.82	3.12	925	C_20_H_34_O_2_	306
6	Ursolic aldehyde	40.93	2.88	880	C_30_H_48_O_2_	440
7	Octadecanoic acid (Stearic acid)	29.88	2.76	861	C_18_H_36_O_2_	284
8	2-Oleoylglycerol	37.62	2.62	849	C_21_H_40_O_4_	356
9	Betulin	43.01	2.20	742	C_30_H_50_O_2_	442
10	Cyclopropanebutanoic acid	25.53	2.07	799	C_25_H_42_O_2_	374
11	Stigmasterol	44.88	1.83	741	C_29_H_48_O	412
12	3,7,12-Trihhdroxycholan-24-oic acid	44.88	1.83	733	C_24_H_40_O_5_	408
13	Coumaran	9.34	1.76	897	C_8_H_8_O	120
14	Methyl o-coumarate	9.34	1.76	871	C_9_H_8_O_3_	164
15	3-O Hexopyranosylhex-2-ul ofuranosyl	18.61	1.65	731	C_18_H_32_O_16_	504
16	Trimethylolpropane ester of ricinoleic acid	37.61	1.55	860	C_21_H_38_O_4_	354
17	2-Methoxy-4-vinylphenol	11.47	1.32	891	C_9_H_10_O_2_	150
18	Cyclopentanone,3-eth3nyl-2,4,4-trimethyl	11.47	1.32	878	C_10_H_16_O	152
19	5-Azecanol	12.89	1.14	709	C_9_H_19_NO	157
20	Cyclohexanol, 1R-4cis-acetamido-5,6cis-epoxy-2tra ns,3cis-dimethoxy-	12.89	1.14	713	C_10_H_17_NO_5_	231
21	Mannose	19.22	1.10	770	C_6_H_12_O_6_	180
22	Desulphosinigrin	19.22	1.10	770	C_10_H_17_NO_6_S	279
23	Diisooctyl phthalate	35.68	0.9	939	C_24_H_38_O_4_	390
24	Cyclohexane, 1R-acetamido-4cis-acetoxy-5,6Zcisep	15.26	0.80	658	C_12_H_19_NO_6_	273
25	(3-Carboxy-3-{[4-Hydroxyt etrahy-dro-2H-pyran-4-yl)methyl] amino}propyl)(dimethyl)sulfonium	15.26	0.80	702	C_12_H_24_NO_4_S	278
26	9-octadecenamide	15.26	0.80	656	C_18_H_35_NO	281
27	Benzeneethanamine, N-(3-methylbutylidene)-	14.32	0.79	886	C_13_H_19_N	189
28	2-Propanamine, N-(phenylmethylene)-	14.32	0.79	689	C_10_H_13_N	147
29	Formamide, N-[1-[(1-Cyanopropyl)hydroxyl-amino]-2-methylepropyle]-	10.36	0.79	678	C_9_H_17_N_3_O_2_	199
30	Ethyl iso-allocholate	45.02	0.69	750	C_26_H_44_O_5_	436

“MF”—match factor, “RT”—retention time.

**Table 2:** In vitro evaluation of the effect of different concentrations of spinach extract compared with negative control on sporulation percentage of *Eimeria tenella* oocysts. The data are expressed as mean ± S.D.

Extract	Concentration	Sporulation (%)		
		After 24 h	After 48 h	After 72 h
Negative control (Pot. dichromate)	2.50%	60.95 ± 1	71.3 ± 0.5	85.43 ± 2
Positive control (formalin)	10%	10.24 ± 1 *	13.12 ± 1 *	18.16 ± 1.2 *
Spinach	10%	27.31 ± 0.9 *	30.81 ± 2.1 *	33.33 ± 2.9 *
	5%	33.35 ± 2.1 *	37.96 ± 1.5 *	40.34 ± 2 *
	2.50%	58.77 ± 2	63.94 ± 1.5 *	69.86 ± 3.2 *
	1.25%	57.28 ± 3.5	67.25 ± 1	73.83 ± 1 *
	0.625%	58.69 ± 2	69.53 ± 1.5	75.16 ± 1 *

(*) The mean difference was significant at *p*-value < 0.001.

**Table 3:** Bloody diarrhea in different groups, as noticed from day 4 to day 10 post-infection.

Day Post-Infection (DPI)							
	4	5	6	7	8	9	10
**Negative Control**	----	----	----	----	----	----	----
**Positive Control**	++	++++	+++	+++	+	+	+
**Diclazuril**	+	++	++	+	----	----	----
**Spinach**	++	+	+	+	----	----	----

(----) None, (+) mild, (++) moderate, (+++) severe, (++++) very severe bloody diarrhea.

**Table 4:** Mortality, survival percentage, and lesion score in different groups throughout the experiment.

Group Name	No. of Deaths	Survival Percentage	Lesion Score
**Negative control**	0	100 *	0
**Positive control**	5	80	3
**Diclazuril**	1	96 *	1
**Spinach**	2	92 *	1

(*) The mean difference was significant at *p*-value < 0.001 after comparing with the positive control group.

**Table 5:** The number of oocysts shed per gram of feces from chickens of different groups from day 4 to day 10 PI.

	Mean Oocyst Count Shed ± SE (per Gram of Feces ×1000)							
	Day Post-Infection (DPI)							
Group	4	5	6	7	8	9	10	Mean ± SE
Negative control	0.00 ± 0.00	0.00 ± 0.00	0.00 ± 0.00	0.00 ± 0.00	0.00 ± 0.00	0.00 ± 0.00	0.00 ± 0.00	0.00 ± 0.00 a
Positive control	0.00 ± 0.00	1.14 ± 0.001	4.15 ± 0.001	4.82 ± 0.006	13.1 ± 0.004	58.7 ± 0.007	10.0 ± 0.058	13.1 ± 0.007 b
Diclazuril	0.00 ± 0.00	0.34 ± 0.001	0.40 ± 0.001	0.67 ± 0.006	3.82 ± 0.004	1.21 ± 0.007	1.1 ± 0.06	1.1 ± 0.007 c
Spinach	0.00 ± 0.00	0.6 ± 0.001	2.28 ± 0.001	3.1 ± 0.006	7.91 ± 0.004	2.95 ± 0.007	2.0 ± 0.058	2.7 ± 0.007 d

Different letters in the same column signify significant differences at *p*-value < 0.05.

**Table 6:** Feed conversion ratio (FCR) was calculated by dividing feed intake (FI) by body weight gain (BWG). Data expressed as mean ± S.D.

	FCR for 1st Week per Bird			2nd Week			3rd Week			4th Week			5th Week		
	BWG g/bird	FI (g)	FCR (g/g)	BWG g/bird	FI (g)	FCR (g/g)	BWG g/bird	FI (g)	FCR (g/g)	BWG g/bird	FI (g)	FCR (g/g)	BWG g/bird	FI (g)	FCR (g/g)
Negative control	65 ± 4.3	96.8	1.49	188 ± 23.7	290	1.55	441.5 ± 24.9 *	640	1.45 **	484.8 ± 20.3 ***	718	1.48 ***	482 ± 51.4 **	733	1.52 ***
Positive control				184 ± 25.4	290	1.56	339.4 ± 20	630	1.86	314.88 ± 16	642	2.04	227 ± 32.5	581	2.56
Diclazuril				188 ± 27.7	290	1.55	381.5 ± 15.6	595	1.56 *	448.96 ± 28.4 **	727	1.62 **	425 ± 67.95 *	701	1.65 ***
Spinach				165 ± 24.9	290	1.76	395.13 ± 10.6	630	1.6 *	450.8 ± 30.8 **	789	1.75 **	390 ± 37.9 *	694	1.78 ***

(*) The mean difference was significant, (**) highly significant, and (***) very highly significant at *p*-value < 0.05.

**Table 7:** Developmental stages of *Eimeria tenella* and goblet cells per 10 villi in ceca dissected on day 8 PI compared with the positive control. Data expressed as mean ± S.D.

Group	Microgamont	Macrogamont	Meront	Developing Oocyst	Total Number of Parasitic Stages	Goblet Cells Number
**Negative control**	0	0	0	0	0	18.3 ± 3.5 **
**Positive control**	5.3 ± 1.5	22.7 ± 7	2.3 ± 0.6	12.7 ± 1.5	43 ± 10.6	11.3 ± 2.5
**Diclazuril**	1.7 ± 0.6 **	2 ± 1 ***	1.3 ± 0.6 *	3.3 ± 1.5 ***	8.3 ± 3.7 ***	15.7 ± 2.1
**Spinach**	1.7 ± 1.2 **	7 ± 1.7 **	3.3 ± 0.6 *	4.7 ± 1.5 ***	16.7 ± 5 **	17.3 ± 3.5 *

The mean difference was significant (*), highly significant (**), and very highly significant (***) at *p*-value < 0.05.

## Data Availability

All related data to this work are available in this manuscript.
